# Targeting Proliferation Signals and the Cell Cycle Machinery in Acute Leukemias: Novel Molecules on the Horizon

**DOI:** 10.3390/molecules28031224

**Published:** 2023-01-26

**Authors:** Andrea Ghelli Luserna di Rorà, Mouna Jandoubi, Giovanni Martinelli, Giorgia Simonetti

**Affiliations:** 1 Biosciences Laboratory, IRCCS Istituto Romagnolo per lo Studio dei Tumori (IRST) “Dino Amadori”, Via Piero Maroncelli 40, 47014 Meldola, Italy; 2 Fondazione Pisana per Scienza ONLUS, 56017 San Giuliano Terme, Italy; 3 Scientific Directorate, IRCCS Istituto Romagnolo per lo Studio dei Tumori (IRST) “Dino Amadori”, Via Piero Maroncelli 40, 47014 Meldola, Italy

**Keywords:** acute myeloid leukemia, acute lymphoblastic leukemia, cell cycle, proliferation, *FLT3*, novel compounds

## Abstract

Uncontrolled proliferative signals and cell cycle dysregulation due to genomic or functional alterations are important drivers of the expansion of undifferentiated blast cells in acute myeloid leukemia (AML) and acute lymphoblastic leukemia (ALL) cells. Therefore, they are largely studied as potential therapeutic targets in the field. We here present the most recent advancements in the evaluation of novel compounds targeting cell cycle proteins or oncogenic mechanisms, including those showing an antiproliferative effect in acute leukemia, independently of the identification of a specific target. Several new kinase inhibitors have been synthesized that showed effectiveness in a nanomolar to micromolar concentration range as inhibitors of FLT3 and its mutant forms, a highly attractive therapeutic target due to its driver role in a significant fraction of AML cases. Moreover, we introduce novel molecules functioning as microtubule-depolymerizing or P53-restoring agents, G-quadruplex-stabilizing molecules and CDK2, CHK1, PI3Kδ, STAT5, BRD4 and BRPF1 inhibitors. We here discuss their mechanisms of action, including the downstream intracellular changes induced by in vitro treatment, hematopoietic toxicity, in vivo bio-availability and efficacy in murine xenograft models. The promising activity profile demonstrated by some of these candidates deserves further development towards clinical investigation.

## 1. Introduction

In hematopoietic cells, proliferation is finely tuned by intrinsic and extrinsic factors regulating the physiological generation of new precursors and the substitution of exhausted cells, while avoiding an uncontrolled cellular growth. In acute leukemias, as in many other cancer types, this balance is broken, leading to the accumulation of proliferating malignant cells that are unable to terminally differentiate into the myeloid or lymphoid lineage. These features are among the well-known hallmarks of cancer [1]. Genetic and functional alterations drive the maintenance of the proliferative and undifferentiated status of blast cells. As such, they represent potential therapeutic targets to selectively kill the malignant cells. The synthesis of novel compounds directed against intrinsic proliferative stimuli or components of the cell cycle machinery executing them is a very active research field in acute myeloid leukemia (AML) and acute lymphoblastic leukemia (ALL). 

In this review, we describe relevant proliferative signals [driven by FMS-like tyrosine kinase 3 (*FLT3*)], molecular and cellular players involved in cell cycle progression/regulation and the related alterations characterizing acute leukemias. Moreover, we present and discuss the advancements made in the last five years (from 2017 until September 2022) through the evaluation of newly synthesized molecules targeting these deregulated processes in acute leukemias.

## 2. Altered Cell Proliferation in Acute Leukemias

### 2.1. FLT3 Alterations in Acute Leukemia: A Driver of Cell Proliferation

**Figure 1:**
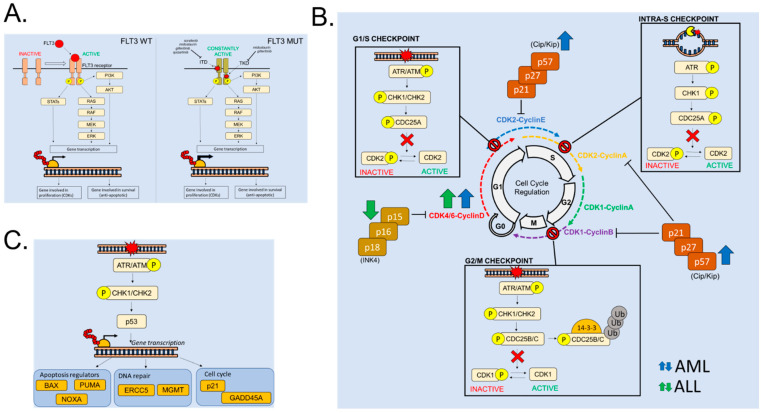
Altered cell proliferation in acute leukemias. (**A**) Schematic representation of the downstream signaling of wild-type (wt, left) and mutated (mut, right) FLT3 receptors. FLT3 inhibitors targeting *FLT3*-ITD and *FLT3*-TKD cells are indicated in the figure. (**B**) Cell cycle regulation in eukaryotic cells. The boxes represent the mechanism of action of the different cell cycle checkpoints. The arrows refer to altered activity (due to functional or genomic aberrations) of different proteins involved in cell cycle regulation found in AML (blue) and ALL (green) patients. (**C**) Role of p53 in the regulation of cell cycle and DNA repair.

Extracellular growth factor receptors or tyrosine kinases (TK) associated with extracellular receptors are frequently over-expressed or mutated in acute leukemia cells. A paradigmatic example is FLT3, a receptor tyrosine kinase able to translate external stimuli into pro-proliferative signaling cascades. It consists of five immunoglobulin-like domains in the extracellular region, a juxtamembrane (JM) domain, a TK domain separated by a kinase insert domain and a C-terminal domain in the intracellular region [2]. The binding of the ligand to the FLT3 receptor has an important role in the regulation of cell proliferation, differentiation and survival through various signaling pathways, including RAS/mitogen-activated protein kinases (RAS/MAPK), Janus kinase/signal transducer and activator of transcription 5 (JAK/STAT5) and phosphatidylinositol 3-kinase/protein kinase B (PI3K/AKT) [3] (Figure 1A). In normal conditions, FLT3 is expressed by early hematopoietic stem cells (HSC) [4], multipotent progenitors [5], common lymphoid and myeloid progenitors [6] and mature dendritic cells [7].

*FLT3* is altered in about one-third of AML patients [3] and, with lower frequency, in ALL cases [8]. *FLT3* represents one of the most frequently mutated genes in AML, with mutations generally belonging to the internal tandem duplication (ITD) or tyrosine kinase domain (TKD) categories. ITDs represent a driver mutation in AML and are located in the JM domain that inhibits receptor auto-activation. *FLT3*-ITD mutations alter this inhibitory effect, leading to an uncontrolled activation [9,10]. *FLT3*-ITD occurs in 20.4% of adult AML patients [11], who are classified as intermediate-risk cases in the absence of adverse-risk genetic lesions, according to the European LeukemiaNet 2022 classification [12]. TKD mutations have been detected in 4.8–7.7% of adult cases [11,13]. This type of mutation also induces aberrant activation of FLT3 signaling [14]. The prognostic impact of *FLT3*-TKD mutations is less clear and these alterations can associate with other mutations and cytogenetic changes [15]. As a consequence of both *FLT3*-ITD and *FLT3*-TKD mutations, the intracellular signals mediated by the TK are constitutively translated, thus promoting the cell cycle progression and, in particular, stimulating the transition from G1 to S phase.

The identification of *FLT3* mutations is highly relevant since it can drive the selection of personalized therapies for AML patients. FLT3 inhibitors can be classified into two groups [16] (Figure 1A). Type I inhibitors (e.g., midostaurin and gilteritinib) interact with the ATP-binding site in the intracellular active pocket of the enzyme and are active against both FLT3-TKD and FLT3-ITD mutated receptors. Type II inhibitors (e.g., quizartinib and sorafenib) bind to the ATP-binding site and interact with an adjacent hydrophobic pocket that is exposed in the inactive conformation and is made inaccessible by TKD mutations. The current therapy of *FLT3*-mut AML relies on the inhibition of FLT3 with midostaurin during induction chemotherapy [17] and with single-agent gilteritinib at relapse [18]. However, patients are prone to the acquisition of secondary mutations conferring resistance to these inhibitors, including, but not limited to, on-target *FLT3* mutations [19]. Several subclones carrying *FLT3*-ITD^D835V/Y/F^ or different TKD mutations alone were responsible for resistance to quizartinib [20], while *FLT3*^N676K^ and *FLT3*^F691L^ gene mutations were associated with resistance to midostaurin [21] and gilteritinib [22] treatment.

### 2.2. Cell Cycle Regulation in Hematopoietic Cells and in Acute Leukemias

The cell cycle is the physiological process promoting cell replication, which is fundamental to sustain tissue growth and repair. In the hematopoietic tissue, the rate of cell division becomes more frequent moving from HSC to differentiated and lineage-committed cells [23,24]. It is well established that HSC are kept in a non-proliferative state, called quiescence, and their proliferation is activated only after specific extracellular stimuli. In all hematopoietic cells, the cell cycle is divided into four consecutive phases characterized by specific events ending with the generation of two daughter cells (Figure 1B). Briefly, the first one is a preparatory synthetic phase (G1) that aims to increase cell size in anticipation of DNA replication (S phase) in which the genome is duplicated. Cells then proceed through a second synthetic phase (G2 phase) to prepare the segregation of the duplicated DNA condensed in twin chromosomes and intracellular organelles to daughter cells (M phase) [25]. The transition from one phase to another is regulated by different multiprotein complexes called cell cycle checkpoints, whose function is to control the proper execution of all steps of each specific phase prior to moving the following one. In eukaryotic cells, four consecutive checkpoints control the transitions through the different phases of the cell cycle and, in particular, the following control steps have been characterized: the G1/S, intra-S, G2/M and mitotic (M) checkpoints [26]. These multi-protein complexes regulate the activity and stability of different Cyclin Dependent Kinase (CDK)–Cyclin complexes whose function is critical to move from one cell cycle phase to the next [25]. Indeed, the core molecular machinery controlling cell cycle progression consists of a family of serine/threonine protein kinases called CDKs [27]. These kinases, representing the catalytic subunits, are activated in most cases by association with regulatory proteins called Cyclins. Cyclins were originally named because their concentration varies in a cyclical fashion during the cell cycle. Thus, their level of expression and, consequently, their availability controls the activity of the different CDK–Cyclin complexes (Figure 1B). The transition throughout the G1 phase is regulated by Cyclin D, whose level starts to grow in the early G1 phase. In this transition, Cyclin D complexes with CDK4 or CDK6 kinases [28]. In HSC, CDK4/6–Cyclin D complexes are crucial as they promote the transition from G0 (quiescence) to G1 phase [29]. The transition from G0 to G1 phase is also regulated by the CDK3–Cyclin C complex [30]. Cyclin E accumulates during G1 phase and is essential, together with CDK2, for the transition from G1 to S phase. The level of Cyclin A starts to increase from the late G1 phase and reaches the highest level at the end of the G2 phase. Cyclin A complexes with CDK2 to promote the transition through the S phase. Lastly, Cyclin B regulates the transition from G2 to M phase as its expression starts to grow from the S phase and reaches the highest level during the G2 phase. To promote mitotic entry, Cyclin B complexes with CDK1, thus generating the mitotic promoting factor (MPF) [28]. 

As mentioned above, the intracellular level of Cyclins controls the activity of CDK–Cyclin complexes. However, additional regulations such as CDK inhibitors (CKIs) and post-transcriptional modifications have been identified. CKIs are families of protein acting, as CDK–Cyclin complexes, in a phase-specific manner and competing with Cyclins for binding with CDKs. Two main groups of CKIs have been identified: the CIP/KIP family and the INK4 family [31,32] (Figure 1B). The first group, composed of p21CIP, p27KIP1 and p57KIP2, acts in multiple phases of the cell cycle by inhibiting: (i) Cyclin D–CDK4/6 activity during the early G1 phase [33]; (ii) Cyclin E–CDK2 during late G1 [32]; (iii) Cyclin A–CDK2 in S phase [33]. Members of the Ink4 family, p16Ink4a, p15Ink4b, p18Ink4c and p19Ink4d, act specifically during the early G1 phase by inhibiting the catalytic activity of CDK4/6–Cyclin D complexes through allosteric competition for binding to Cyclins [32]. As already mentioned, CDK–Cyclin complexes are regulated by post-transcriptional modifications as a consequence of cell cycle checkpoint activation. Indeed, in the event of DNA damage or replicative stress, different intracellular cascades promote the phosphorylation or the dephosphorylation of CDK complexes, thus inducing their activation or inactivation. In the case of DNA damage, different kinases involved in the DNA damage response (DDR) pathway directly or indirectly promote the inhibition of CDK–Cyclin complexes. The function of DDR pathways is to inhibit cell cycle progression until the DNA damage is repaired by specialized pathways. Eukaryotic cells have evolved different DDR pathways to respond to different types of DNA damage and depending on the specific cell cycle phase in which the damage has been detected. During S phase, replicative stress causing stalled replicative forks or single-strand breaks (SSBs) of the DNA activates the ATR-CHK1 pathway. On the other hand, double-strand breaks (DSBs) of the DNA structure activate the ATM-CHK2 pathway that acts on the transition from S, G2 and M phases. Following an intracellular cascade, the ATR/ATM kinases activate their downstream target cell cycle checkpoint kinase 1 (CHK1) and 2 (CHK2), respectively (Figure 1B). After their activation, CHK1 and CHK2 promote the phosphorylation of several target proteins and, in particular, of different phase-specific phosphatases called CDC25A/B/C [34,35]. This final event indirectly regulates the activity of CDKs. Indeed, the three phosphatases remove the inhibitory phosphorylations from CDKs, leaving them available for Cyclin binding. In the transition through S phase, the CDC25A phosphatase removes the inhibitory phosphorylation on CDK2 (Thr14 and Tyr15) promoting the generation of CDK2–Cyclin E/A complexes. In the presence of DNA damage, CHK1 and CHK2 kinases phosphorylate CDC25A (Ser136), thus promoting its ubiquitination and proteasomal degradation. As a consequence, the inhibition of CDC25A causes an S-phase delay. Similarly, CDC25B and CDC25C regulate the transition from G2 to M phase by modulating the activity of CDK1 and, in particular, by removing the inhibitory phosphorylation at two sites (Thr14 and Tyr15). During DDR, CHK1 phosphorylates CDC25B (Ser323) and promotes its binding to 14-3-3, thus blocking its catalytic activity [36]. CDC25C activity is also regulated by phosphorylation (on Ser216) induced by both CHK1/CHK2 kinases. The events that follow CDC25C phosphorylation are similar to those following CDC25A phosphorylation and end with binding to 14-3-3 and its functional inhibition. The degradation of CDC25C impairs CDK1–Cyclin B activation and consequently compromises the transition through the G2/M phase. Lastly, a central role in the regulation of the cell cycle and DNA repair is played by the guardian of the genome, the tumor suppressor p53. Following the identification of DNA damage, p53 is activated by phosphorylation in an ATM/CHK2- or ATR/CHK1-dependent manner. p53 then promotes the transcription of several target proteins, including apoptotic regulators (e.g., PUMA, NOXA and BAX), DNA repair elements (e.g., ERCC5 and MGMT) and cell cycle regulators (e.g., p21Cip1 and Gadd45) (Figure 1C). The p53–p21-dependent response to DNA damage mediates different effects on cell cycle progression: (i) p21Cip1 interferes directly with CDK–Cyclin complexes involved in the transition from the G1 to the S phase; (ii) p53 inhibits the transcription of genes involved in the G2/M checkpoint such as the Cyclin B1 gene (CCNB1). In summary, the activation of the p53–p21 pathway promotes a robust G1 phase arrest [37,38].

### 2.3. Cell Cycle Alterations in Acute Leukemia

Alterations in the functionality of key factors involved in cell cycle regulation can lead to severe consequences, such as malignant transformation. Dysregulation of CDKs and associated Cyclins is frequently observed in hematological malignancies. CDK6 is predominantly expressed in the hematopoietic compartment [39] and loss of CDK6 results in impaired generation of several blood cell types [40]. As an opposite mechanism, CDK6 hyperactivation promotes cell proliferation in ALL and lymphoma patients [41,42] (Figure 1B). In acute leukemia patients, CDK6 over-expression has been frequently associated with *KMT2A* translocations, as it has been demonstrated that CDK6 is a direct target of *KMT2A* fusion proteins [43]. Moreover, CDK6 can be activated by Cyclin D2 and D3 upregulation mediated by *FLT3*-ITD in AML [44]. In addition to CDK/Cyclin functional dysregulations, cancer cells are frequently characterized by molecular alterations in different CKIs (Figure 1B). Indeed, the *CDKN2A*/*B* gene locus, located at chromosome 9p21, is one of the most frequently deleted, mutated and epigenetically silenced sites in cancer cells. In ALL patients both p16INK4a and p15INK4b are frequently deleted [45]. In particular, T-ALL is frequently associated with p16INK4a loss, while p15INK4b deletions are more often observed in pediatric ALL [46]. In mouse models of *BCR*-*ABL1*-positive ALL, the deletion of *p19ARF* initiates a more aggressive disease and confers resistance to the kinase inhibitor imatinib [47]. Regarding the CIP/KIP family, inactivating mutations or deletions of p21Cip1/Waf are very rare in acute leukemias. Conversely, he over-expression of p21Cip1/Waf has been found in AML patients expressing an *AML1*-*ETO* fusion transcript [48]. Regarding p27Kip1, its levels of expression correlate with the prognosis of different hematological malignancies. For instance, high p27Kip1 levels are associated with increased disease-free survival in AML [49]. Moreover, Cell division cycle 20 homologue (CDC20), which is involved in mitotic progression, is frequently over-expressed in acute leukemias [50,51], as well as in other blood and solid tumors [52,53].

## 3. Novel Molecules Targeting Proliferative Mechanisms in Acute Leukemias 

### 3.1. Novel Molecules Targeting FLT3

**Figure 2:**
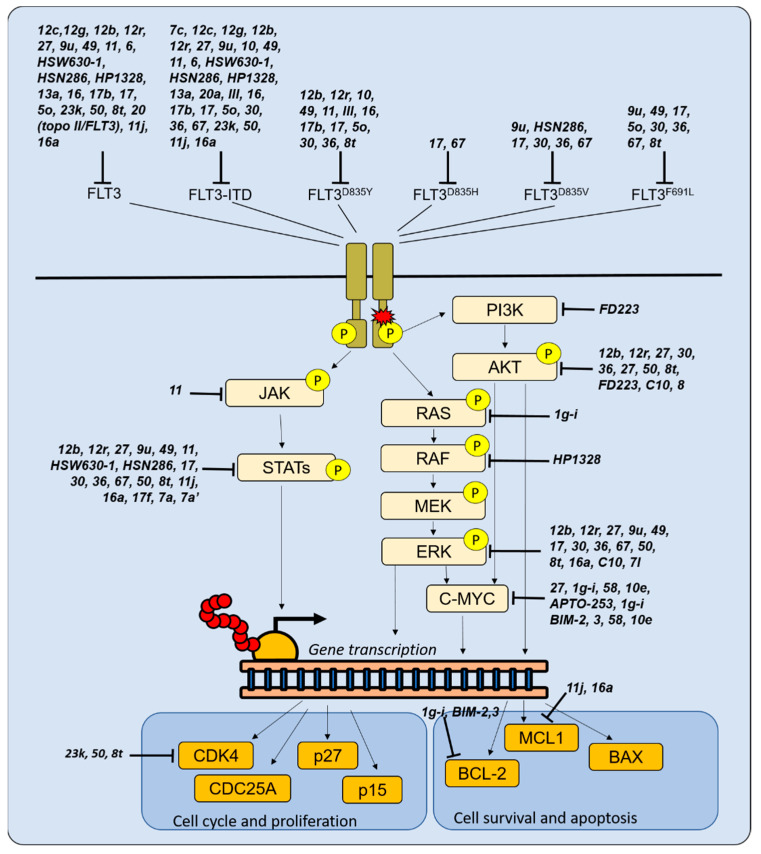
Novel molecules targeting proliferative mechanisms in acute leukemias. In the picture the different molecules targeting proliferative mechanisms presented in the manuscript are reported in italics.

Given the high frequency of *FLT3* mutations and the development of resistance to drugs targeting them, there is a continuous interest in novel FLT3 inhibitors (Table 1 and Table 2 and Figure 2) to be used in combination therapies (e.g., through multi-targeted approaches), with the aim of eradicating leukemia while decreasing toxicity and enhancing treatment tolerability. Some of them have also been shown to induce cell cycle arrest (Table 2).

A 5-(4-fluorophenyl)-N-phenyloxazol-2-amine (compound **7c**) inhibited the proliferation and increased apoptosis of *FLT3*-ITD cells in vitro and reduced the in vivo growth of tumors obtained by xenotransplantation of the *FLT3*-ITD MV4-11 AML cell line, without causing obvious toxicities [54]. Similar to other FLT3 inhibitors [80], compound **7c** induced downregulation of DDR genes belonging to the homologous recombination (BRCA1, BRCA2, BARD1 and RAD51) and non-homologous end joining (XRCC4, XRCC5 and XRCC6) pathways and accumulation of DNA damage [54]. As a consequence, compound **7c** sensitized *FLT3*-ITD AML cells to treatment with the PARP inhibitor olaparib [54,81].

4-((6,7-dimethoxyquinoline-4-yl)oxy)aniline derivatives possessing the semicarbazide moiety as the linker (in particular compound **12c** and **12g**) were active as FLT3 inhibitors and reduced the growth of MV4-11 and HL-60 cells [55]. 4-azaaryl-N-phenylpyrimidin-2-amine derivatives (compounds **12b** and **12r**) inhibited the activity of FLT3 kinase, FLT3-ITD and other mutant forms and of the downstream signaling cascade (STAT5, ERK, AKT, S6RP and eIF4E) and induced apoptosis [56]. They displayed an antiproliferative specificity for *FLT3*-ITD cells over those expressing the wild-type (wt) kinase or even lacking FLT3 expression.

An important feature in the development of FLT3 inhibitors is kinase selectivity, which is directly related to off-target toxicity. Indeed, several novel compounds showed a specific anti-FLT3 activity, but inhibited a spectrum of additional kinases.

N-(4-(6-Acetamidopyrimidin-4-yloxy)phenyl)-2-(2-(trifluoromethyl)phenyl)acetamide (CHMFL-FLT3-335, compound **27**) showed a specific anti-leukemic activity against *FLT3*-ITD AML models (MOLM-13, MOLM-14, MV4-11 AML cell lines) when compared with other AML subtypes [68]. However, it showed a poor activity against TKD mutations. Compound **27** also inhibited PDGFRβ, HPK1, CSF1R, PDGFRα and c-KIT (though to a lower extent compared with *FLT3*-ITD). It reduced the phosphorylation levels of the downstream signaling pathway mediators STAT5, AKT and ERK, and also the expression of the c-MYC oncogene. Treated cells were arrested at the G0/G1 phase of the cell cycle and underwent apoptosis. The anti-leukemic effect was confirmed in primary AML cells expressing *FLT3*-ITD and in xenograft models obtained by MV4-11 cell inoculation, by a reduction in tumor growth. The same in vitro and in vivo effects were also observed by treating AML cell lines with pyrrolo[2,3-d]pyrimidine derivatives, an in particular compound **9u** [69]. Notably, this molecule was also active against *FLT3*-ITD^D835V^ and *FLT3*-ITD^F691L^ cells. The ability to target *FLT3* mutations arising as a mechanism of resistance represents another relevant feature in the development of novel compounds, as it allows us to answer a clinical need.

A 8,9,10,11-tetrahydro-3H-pyrazolo[4,3-a]phenanthridine (HSD1169, compound **10**) was active against MV4-11, MOLM-14 cell lines and MOLM-13 harboring the *FLT3*-ITD^D835Y^ mutation by inducing cell cycle arrest at the G1 phase [70]. The compound also inhibited the activity of LRRK2, MELK, DYRK1B, CLK1, MNK2, TOPK, ROCK2, MSK2, p70S6K, PKG1a and CDK2–Cyclin A1 [71] and decreased the expression of TOPK, a kinase collaborating with FLT3 that is highly expressed in AML [82]. A derivative of compound **10**, obtained by substitution of position 1 of the 3H-pyrazolo[4,3-f]quinoline core (compound **49**), showed a higher selectivity towards FLT3 proteins [71]. It reduced the phosphorylation level of STAT5 and ERK1/2 signaling proteins, induced cell cycle arrest at the G1 phase, activated the apoptotic cascade and inhibited leukemia growth in a mouse-disseminated AML model.

Among flavones and flavonols, an *O*-methylated flavonol (compound **11**) showed an elevated cytotoxic activity against acute leukemia cells [72]. In particular, it was selectively more active against the AML MOLM-13, MV4-11, HL-60 and T-ALL MOLT-4 models compared with solid cancer cell lines. It favored the accumulation of cells in the G0/G1 phase of the cell cycle and induced apoptosis. The molecule reduced the levels of the phosphorylated forms of FLT3, RSK2, STAT3, eIF4E and p53, and also exhibited inhibitory activity against JAK2, JAK3, MNK2 and NTRK2, as observed by kinase profiling. Moreover, compound **11** inhibited the activity of FLT3-ITD and FLT3^D835Y^. Additional flavonoids and their derivatives were also shown to target FLT3 (including its mutated forms) [73] or MNKs [83], resulting in cell apoptosis and G0/G1 cell cycle arrest.

Semisynthetic derivatives of fradcarbazole A (and in particular compound **6**) also inhibited FLT3 kinase along with c-KIT and the cell cycle protein CDK2, while inducing arrest of the cell cycle at the G0/G1 phase and apoptosis of MV4-11 cells [74]. Notably, these compounds were not active against normal peripheral blood mononuclear cells (PBMCs), with IC50 values greater than 10 μM.

Ma et al. showed that 3-aminoisoquinoline analogs (HSW630-1 and its analogs) are potent FLT3 inhibitors [57]. They reduced the growth of MV4-11 and MOLM-14 AML cells and the phosphorylation of FLT3 and STAT5. Moreover, they also displayed an activity against c-KIT and TRKC enzymes. Starting from these molecules, the group synthesized novel amino isoquinoline, quinoline or quinazoline compounds [58]. In particular, HSN286 and its analogs inhibited both FLT3 and downstream SRC-family kinases, leading to reduced phosphorylation of FLT3, STAT5, STAT3 and p38. This approach could represent a valuable strategy to inhibit cell proliferation [84] even in the event of *FLT3* mutations.

A derivative of N-(3,4-dimethoxybenzyl)-1-phenyl-1H-benzimidazol-5-amine (compound HP1328) was designed as an FLT3-ITD inhibitor with good aqueous solubility [59]. The compound induced apoptotic cell death of Ba/F3 expressing FLT3-ITD, MOLM13 and MV4-11 in a dose-dependent manner and of primary *FLT3*-ITD AML cells. Moreover, it potentiated the cytotoxicity of the anthracycline idarubicin. HP1328 activity has been confirmed in xenograft models, by improvement of the survival of leukemia-bearing mice. The treatment was suitable for oral administration and well tolerated. The kinase profile of inhibitory effects caused by HP1328 revealed a substantial selectivity over potential off-target kinases as BRAF, VEGFR and FGFR. However, the molecule selectively targeted c-KIT, thus limiting its further development due to the in vivo induction of myelosuppression.

Since c-KIT inhibition is largely responsible for myeloid immunosuppression, a number of studies have focused on newly synthesized molecules able to selectively target FLT3 and its mutant forms over c-KIT. Among them, the pyrimidine-4,6-diamine derivative compound **13a** inhibited the growth of MOLM-14 and MV4-11 AML cell lines and of Ba/F3 cells expressing FLT3-ITD by accessing the FLT3 allosteric pocket while sparing c-KIT [60]. However, this compound was not effective against the *FLT3*^D835Y^ mutation.

Novel amino-acid-substituted sunitinib analogues released active compound candidates have been developed in order to improve selectivity towards FLT3 [61]. Among them, the mono-carboxylic acid compound **20a** provided the best results in terms of affinity against FLT3-ITD and activity against MV4-11 cells. 

Promising results have been achieved with phenylethenylquinazoline derivatives in terms of selectivity [62]. They inhibited the activity of FLT3-ITD, FLT3^D835Y^ and the quizartinib-resistant FLT3-ITD^D835Y^ double-mutant enzyme while exerting almost no effect over c-KIT. Compound **III**, the most promising derivative, inhibited cell growth while inducing apoptosis in vitro, and effectively reduced the tumor size while being well-tolerated by murine xenograft models.

Derivatives of bis(1H-indol-2-yl)methanones, and in particular compound **16** and its carbamate **17b**, selectively inhibited (among 249 kinases) FLT3, RET and ZAK [63], the latter being involved in the TGFβ/JNK pathway [85] and in the regulation of the cell cycle [86]. These compounds were effective against FLT3-ITD and FLT3^D835Y^ mutant proteins; they inhibited the growth and induced apoptosis of MV4-11, MOLM-13 and EOL-1 cells [63], which shared *KMT2A* alterations (rearrangements or partial tandem duplication) associated with differentiation blockade, and *FLT3* aberrations (EOL-1 was characterized by FLT3 overexpression [87] and mutant *PDGFRA* [88]). in vivo studies revealed a good tolerability of compound **17b** [63].

A (Z)-N-(5-((5-Fluoro-2-oxoindolin-3-ylidene)methyl)-4-methyl-1H-pyrrol-3-yl)-3-(pyrrolidin-1-yl)propanamide (compound **17**) showed promising in vitro and in vivo properties as an FLT3 inhibitor [64]. The molecule inhibited the proliferation of MV4-11 and MOLM-13 cells and was active against the FLT3^D835Y/D835H/D835V^ and FLT3-ITD^D835Y/D835V/F691L^ mutant forms. It reduced the phosphorylation of the downstream signaling pathway mediators STAT5 and ERK and induced cell cycle arrest in the sub-G1 phase and apoptosis, while displaying a very low activity on *FLT3*-wt AML cells and other cancer cell lines. Compound **17** had a favorable pharmacokinetic profile with high oral bioavailability and the mechanism of action has been confirmed in murine xenograft models obtained by AML cell line transplantation that also showed low levels of treatment toxicity. Notably, compound **17** was able to reduce the growth of sunitinib- and quizartinib-resistant cells. 

Zhang et al. synthesized and tested imidazo[1,2-a]pyridine thiophene compound inhibitors of FLT3 and its mutant forms [65]. Among the tested molecules, compound **5o** was effective against MOLM-14 AML cells, even when manipulated to express the mutant FLT3-ITD^D835Y^ or FLT3-ITD^F691L^ forms. Moreover, compound **5o** was more potent than the FLT3 inhibitor quizartinib and was not cytotoxic against Ba/F3 normal pro-B cells.

On the same line of reasoning, 2-aminopyrimidine derivatives were synthesized with the aim of selectively inhibiting *FLT3*-ITD and its mutant forms while sparing the c-KIT kinase, thus reducing the myelosuppressive effect [66]. Compounds **30** and **36** inhibited the growth of MV4-11 cells and of Ba/F3 cells engineered to express FLT3-ITD, FLT3^D835V/F^, FLT3^F691L^, FLT3-ITD^F691L^ and FLT3-ITD^D835Y^. The compound showed good liver microsomal stability, poor antiproliferative effect against *FLT3*-wt tumor cells, low toxicity to normal cells and weaker inhibitory activity against c-KIT compared with the FLT3 inhibitor gilteritinib in a zebrafish model. The molecules reduced FLT3 phosphorylation, thus affecting the downstream pathway, as revealed by reduced activation of STAT5, AKT and ERK. Moreover, they exerted an inhibitory activity against additional kinases including TRKA and Aurora A. The compound’s activity was confirmed in the murine xenograft model obtained by transplantation of MV4-11 cells by inhibition of the tumor growth rate and in mice inoculated with Ba/F3 expressing FLT3-ITD^D835Y^ by prolongation of animal survival.

Starting from the observation that new compounds with pyrazole amine scaffolds exhibited potent inhibitory activity and selectivity against *FLT3*-ITD AML cells [89], novel 3-amine-pyrazole-5-benzimidazole compounds were designed and tested in order to overcome secondary resistance mutations [75]. Among them, compound **67** exerted an antiproliferative activity not only against *FLT3*-ITD cell lines, but also against Ba/F3 *FLT3*-ITD^D835V^, Ba/F3 *FLT3*-ITD^F691L^ and Ba/F3 *FLT3*-ITD^Y842H^. It effectively induced apoptosis and cell cycle arrest at the G1 phase by blocking the phosphorylation of FLT3, STAT5 and ERK. Compound **67** inhibited tumor growth in xenograft models (by intravenous injection due to poor oral bioavailability), while showing limited hematological and systemic toxicity.

To potentiate the anti-leukemic activity, novel molecules with a dual specificity were also synthetized. Indeed, monotherapies against one single target have shown limited efficacy in acute leukemias, due to the rapid development of escape strategies by malignant cells. For example, activation of RAS/MAPK signaling through functional [90] or genomic alterations [19,22] is a common mechanism of resistance to inhibitors of FLT3 or the antiapoptotic protein BCL-2, which are currently used in the treatment of AML. Therefore, co-targeting of two leukemogenic pathways may provide improved therapeutic benefits while sparing off-target toxicities.

Li et al. presented a series of 6-(pyrimidin-4-yl)- 1*H*-pyrazolo[4,3-*b*]pyridine derivatives that inhibited FLT3 and CDK4 kinases [76]. Some of them were more effective than the selective CDK4/6 inhibitor Abemaciclib and compound **23k** showed the highest potency. Molecular docking studies showed that **23k** could form a relatively stable protein–small-molecule complex by binding to both CDK4 and FLT3. The molecule induced cell cycle arrest in the G0/G1 phase and apoptosis in a concentration-dependent manner and inhibited phosphorylation of the downstream CDK2/4 factor RB and of FLT3 in the MV4-11 AML cell line. Compound **23k** was also tested in vivo in the MV4-11 xenograft tumor model and reached a 67% tumor growth inhibition rate at a high dose, suggesting poor oral bio-availability.

Similar results were obtained by testing a series of 1-H-pyrazole-3-carboxamide derivatives, acting as FLT3 and CDK inhibitors [77]. Among them, compound **50** potently inhibited FLT3, CDK2, CDK4 and CDK6, resulting in the suppression of phosphorylation of RB, FLT3, ERK, AKT and STAT5, cell cycle arrest at the G0/G1 phase, induction of apoptosis and in vivo activity in AML. Aiming to improve the FLT3/CDK inhibitory activity, novel 1H-pyrazole-3-carboxamide derivatives were also developed from compound **50 [67].** Compound **8t** had a multi-kinase inhibitory capacity, by targeting KDR/VEGFR2, ERK7, FLT1, FLT4 and GSK3 in addition to FLT3 and CDKs, that was responsible for its broad activity against acute leukemia but also solid tumor models. Moreover, it also exerted an inhibitory activity against a variety of FLT3 mutants. At the intracellular level, compound **8t** blocked the phosphorylation of STAT5/AKT/ERK and of RB.

Novel pyrido-dipyrimidines showed activity towards topoisomerase II and/or FLT3, which was confirmed by docking studies [78] and successfully combined in the dual activity of compound **20**. Biologically, this molecule induced upregulation of p53 and TNFα, accumulation of cells in the G2/M phase of the cell cycle and apoptosis in the HL-60 cell line.

A series of N-phenyl-4-(thiazol-5-yl)pyrimidin-2-amines and 4-(indol-3-yl)-N-phenylpyrimidin-2-amines was investigated with the aim of achieving dual inhibition of FLT3 and MNK2 as a potential strategy to avoid resistance mediated by the activation of parallel signaling pathways [79]. Indeed, MNK kinases act as downstream effectors of MAPK pathways mediating the phosphorylation of eIF4E, a protein that plays a key role in the regulation of mRNA translation, and counteract chemotherapy-induced antileukemic responses [91]. Three molecules were selected for their promising activity as FLT3 (**11j**), dual (**16a**) or MNK2 (**16h**) inhibitors [79]. They all showed a selective activity against their target(s) over CDK2, B-RAF MAPKs, p38a, PI3K, AKT and mTOR. In the MV4-11 AML model, **11j** decreased STAT5 phosphorylation, **16h** inhibited eIF4E phosphorylation and showed off-targets effects over STAT5, while **16a** reduced phosphorylated STAT5, ERK, p38 and eIF4E. Both **11j** and **16a** compounds arrested the cells in the G1 phase of the cell cycle in a dose-dependent manner and induced apoptosis along with downregulation of the pro-survival protein MCL-1.

### 3.2. Targeting Other Proliferative Signals in AML

In addition to FLT3 inhibition, targeting of other proliferative signals, including kinases (e.g., PI3Kδ and STAT5) and oncogenic mechanisms (e.g., c-MYC), has raised interest in recent years (Table 3 and Figure 2). 

A PI3Kδ inhibitor (FD223, compound **13**) was obtained by bioisosteric replacements based on an indole scaffold [92]. The compound inhibited the proliferation of several AML cell lines by suppressing AKT phosphorylation, thus arresting the cell cycle at the G1 phase and inducing apoptosis. Pharmacokinetic studies revealed an acceptable oral bioavailability and in vivo experiments showed a dose-dependent tumor growth inhibition, with induction of tumor necrosis, decreased expression of Ki67 and no signs of acute toxicity.

Among the mediators of oncogenic signals in AML, STAT5 plays an important role in multiple disease subtypes [101,102]. PPARα/γ ligand derivatives, and in particular compound **17f**, which is characterized by a free nitrogen on the indole ring along with a C-6 tetrahydroquinoline 3-pyridinyl substitution, selectively inhibited STAT5 activity, resulting in an antiproliferative effect against AML cellular models [93]. The compound also restored sensitivity to cytarabine in resistant AML cells through the inhibition of STAT5B but not STAT5A protein expression [103]. The **7a** and **7a’** analogs of **17f** showed a higher potency as inhibitors of STAT5 activity and expression, resulting in the downregulation of its targets *PIM1* and *CISH* [94]. Both positions of the pyridinyl moiety on the tetrahydroquinoline aromatic part and its nitrogen position were essential in the antileukemic properties of **7a** and **7a’**.

Interest has been raised in recent years in the development of molecules able to bind G-quadruplexes, noncanonical nucleic acid structures found in specific guanine-rich DNA or RNA sequences, including the promoter regions of the *c-MYC*, *K-RAS* and *c-KIT* oncogenes and of the anti-apoptotic gene *BCL-2*. The stabilization of G-quadruplex structures can be therapeutically exploited to attenuate the transcription of pro-tumorigenic genes, leading to an antiproliferative effect [104]. Treatment of AML cells with the small-molecule 2-indolylimidazole[4,5-d]phenanthroline derivative APTO-253 [105] induced CDKN1A expression, G0/G1 cell cycle arrest, and apoptosis through stabilization of G-quadruplex DNA, inhibition of MYC expression and induction of DNA damage [106]. APTO-253 also entered clinical investigation for patients with relapsed/refractory AML or myelodysplastic syndrome (#NCT02267863). However, its clinical development has been stopped by the company. In the meantime, the development of novel heterocycles binding to DNA G-quadruplexes has led to the synthesis of a series of new substituted 2,9-bis [(substituted-aminomethyl)phenyl]-1,10-phenanthroline derivatives, showing a selective activity against human leukemia cells over normal PBMCs [107]. This was the starting point for the synthesis of a new series of 2,9-bis[(substituted-aminomethyl)phenyl]-1,10-phenanthroline derivatives [95]. Among them, compounds **1g**–**i** provided the most promising results in terms of activity against AML cell lines, stabilization of c-MYC, BCL-2 and K-RAS promoter G-quadruplexes and inhibition of telomerase activity.

The biimidazole derivative BIM-2 also showed a promising anti-leukemia activity as a dual c-MYC/BCL-2–quadruplex binder [96]. It reduced the expression of both c-MYC and BCL-2 and triggered cell cycle arrest at the G0/G1 phase and apoptosis in AML cells, thus showing a promising example of dual targeting, with activity against signals supporting both proliferation and survival of leukemic cells.

c-MYC downregulation was also a consequence of quinizarin derivative treatment in T-ALL cells [97]. Quinizarin quaternary ammonium salt **3** inhibited the growth of AML and T-ALL cell lines. However, it displayed a similar toxicity against normal human embryonic kidney HEK-293 cells. The compound led to G0/G1 phase arrest with a decreased S phase cell population, induction of apoptosis and accumulation of intracellular reactive oxygen species. Although the direct targets of compound **3** were not investigated, the treatment also triggered the downregulation of BCL-2 and NOTCH1.

Aiming at c-MYC inhibition, Feng et al. synthesized a series of 3,5-dimethylisoxazole derivatives targeting the epigenetic reader bromodomain-containing protein 4 (BRD4). In particular, compound **58** effectively inhibited the proliferation of AML cell lines by causing a reduction in c-MYC protein levels, cell cycle arrest in G1 and apoptosis [98]. Structure–activity relationship (SAR) and docking studies revealed that the 3-hydroxyisoindolin-1-one chemical scaffold could also act as BRD4 inhibitor [99]. Among the synthesized 3-Hydroxyisoindolin-1-one derivates, compound **10e** displayed antiproliferative properties against AML cell lines through c-MYC downregulation and activation of the intrinsic apoptotic pathway. In addition to BRD4, the bromodomain and PHD finger-containing (BRPF) family has been studied as a potential therapeutic target in AML. Indeed, BRPF1 is highly expressed in acute leukemia cell lines [108] and sustains the expression of HOX genes [109], which play a pathogenic role in diverse AML subtypes [110,111,112,113]. A series of Quinolin-2-one Hits has been synthesized and tested in order to achieve selective inhibition of BRPF proteins. Among them, **13-d** was experimentally confirmed as a potent inhibitor of BRPF1 [100]. The compound exerted an antiproliferative activity on AML cell lines and displayed an acceptable oral bioavailability according to pharmacokinetic studies conducted in mouse models. Recently, aiming to achieve a potent anti-leukemic activity, novel dual HDAC8/BRPF1 (sulfonamide derivatives **23a-b** of an HDAC8 inhibitor) and HDAC6/BRPF1 (compound **37**) inhibitors were synthesized [114] starting from benzhydroxamic acids, and they proved to be potent and selective HDAC8 inhibitors [115,116]. However, they demonstrated a poor cellular activity [114].

### 3.3. Novel Compounds with Unknown Targets That Inhibit Acute Leukemia Cell Proliferation 

A group of novel compounds showing an antiproliferative activity against acute leukemia cells remains to be characterized in terms of target specificity (Table 4).

A 3′,5′-diprenylated chalcone belonging to the flavone family, (E)-1-(2-hydroxy-4-methoxy-3,5-diprenyl)phenyl-3-(3-pyridinyl)-propene-1-one (**C10**), inhibited the proliferation and induced apoptosis of the human erythroleukemia cell line HEL in a time- and dose-dependent manner [117]. The molecule caused a downregulation of the transcription factor Friend Leukemia Integration 1 (FLI-1) in HEL cells and a reduction of the phosphorylation levels of p38/MAPK and ERK1/2, suggesting an activity on the MAPK pathway, supporting cell proliferation (Figure 2). A potential effect on cell autophagy has been also hypothesized, based on the inhibitory effect exerted on the AKT/mTOR pathway (Figure 2).

The MAPK pathway has been also inhibited by a dithiocarbamate ester of parthenolide (compound **7l**) in AML [118]. The molecule induced apoptosis and suppressed the clonogenic capacity of primary leukemia stem cells while sparing normal cells from healthy donors. Moreover, it prolonged the survival of AML-patient-derived xenograft mice.

In addition to dithiocarbamate esters of parthenolide, neglectin derivatives were active against AML models [119]. Compound **8**, carrying an N,N-dimethylamino ethoxyl moiety at the C-6 position, induced cell cycle arrest at the G0/G1 phase in HL-60 cells by suppressing the activation of AKT (Figure 2) and S6K1, a serine/threonine kinase belonging to the mTORC1 pathway. A new 2,4-dinitrobenzenesulfonamide derivative (S1) triggered G0/G1 blockade, intrinsic apoptosis and reduction of Survivin expression in the Jurkat T-ALL model, while showing no cytotoxicity towards normal erythrocytes and mononuclear cells [120]. Analogs based on the fungi-derived polyenylpyrrole products rumbrin and auxarconjugatin-B (and in particular the octatetraenylpyrrole analog **3s**) were also able to suppress the proliferation of T-ALL cells [121].

### 3.4. Novel Molecules Targeting the Cell Cycle Machinery

Starting from the observation that functional alterations of the cell cycle machinery occur in acute leukemias and the expression of some of its components is deregulated, the cell cycle and DDR pathway have become appealing targets for the development of anti-leukemic therapies (Table 5).

Thirty 2-carbomethoxy-3-substituted indoles have been synthetized and tested against acute leukemia cells [122]. Among them, compound **20** showed a selective anti-leukemic activity while sparing healthy T lymphocytes up to a dose of 100 μM. The molecule induced cell cycle arrest at G2/M and apoptosis in HL-60 cells, CCRF-CEM and RS4;11 T-ALL and B-ALL models, respectively. Mechanistically, its activity has been linked to microtubule depolymerization, as supported by the observation of reduced tubulin expression, abnormal chromosomal segregation and fragmented centrosomal material. Accordingly, gene expression profiling highlighted enrichment of gene sets involved in the G2/M checkpoint, the mitotic spindle and NOTCH signaling and a decrease in the cholesterol homeostasis in treated HL-60 cells. Moreover, compound **20** induced differentiation of HL-60 cells, as revealed by upregulation of the CD11b and CD14 myeloid markers. Of note, the treatment improved the survival of mice engrafted with RS4;11 leukemic cells.

Induction of microtubule depolymerization was also the mechanism of action of (Z)-2-((2-((1-ethyl-5-methoxy-1H-indol-3-yl)methylene)-3-oxo-2,3-dihydrobenzofuran-6-yl)oxy)acetonitrile (**5a**) and (Z)-6-((2,6-dichlorobenzyl)oxy)-2-(pyridin-4-ylmethylene)benzofuran-3(2H)-one (**5b**), which showed a selectivity for the colchicine-binding site on tubulin [123]. The molecules inhibited the growth of T- and B-ALL cell lines while being less effective against normal B-lymphoblast cells and blocked disease progression in a *myc*-induced T-ALL zebrafish model.

With the aim of targeting the mitotic checkpoint, we have designed a series of tryptamine derivatives [127]. Compound **9**, which is characterized by 2-aminopyrimidyl- and trichloroethyl- moieties, similarly to those found in Apcin [128], a small molecule that blocks the interaction between the anaphase-promoting complex/cyclosome (APC/C) and CDC20 [53], showed a selective activity against acute leukemia models over solid tumors. Indeed, it inhibited the growth of MV4-11 AML cells, the REH B-ALL model and especially the T-ALL cell line Jurkat-6. Notably, the pyrimidine ring and aminal nitrogens, together with the hydrophobic trichloromethyl group, were identified as important structural motifs for Apcin activity SAR experiments. Future studies are needed to define the intracellular activity of compound **9**.

Induction of blast cell differentiation is emerging as a promising strategy, not only against acute promyelocytic leukemia, but also in the AML field [129]. A CDK2-targeted proteolysis-targeting chimera (PROTAC) has been developed to selectively degrade CDK2 while sparing the other CDKs [124]. The molecule (CPS2) exerted an antiproliferative effect (also favored by the downregulation of Aurora Kinase A), induced remarkable differentiation of AML cell lines and primary cells and arrested leukemic cell growth in vivo. Moreover, CPS2 sensitized the cells to CDK4/6 or PI3K inhibition and enforced the induction of differentiation by all-trans retinoic acid. The compound altered the expression of genes involved in myeloid leukocyte differentiation and activation, and cytokine-mediated signaling pathways and some of them were not altered by inhibition of CDK2 enzymatic activity, thus confirming the importance of degrading the molecule in order to achieve a stronger effect on cell differentiation.

As highlighted in the previous paragraphs, one key step in the cell cycle is the maintenance of DNA integrity. CHK1 is a central component in the DDR. Diaminopyrimidine derivatives (and in particular compound **13**) were able to inhibit its activity, as demonstrated by inhibition of its autophosphorylation [125]. Compound **13** suppressed the proliferation of MV4-11 AML cells, while exhibiting low inhibitory potency against normal PBMCs. Moreover, the molecule also displayed a moderate oral bioavailability in vivo, suggesting its suitability for further studies on murine models.

Novel 1-substituted pyrido[4,3-b]carbazole derivatives of olivacine [130] also exerted a role in the maintenance of DNA integrity in leukemic cells [126]. Indeed, they forced the expression of p53 and p21Cip1 proteins in the *TP53*-mutant CCRF-CEM T-ALL model, thus causing a decrease in the number of cells in the S phase of the cell cycle and the induction of cell death via apoptosis. Notably, several attempts have been made in recent years in the development of novel molecules and strategies to restore the functionality of p53 in cancers [131], which may in the future answer an unmet clinical need in acute leukemias.

## 4. Conclusions

We have here provided a comprehensive review of recently synthesized compounds targeting the cell cycle or cell proliferation in AML and ALL. They include microtubule-depolymerizing or p53-restoring agents, G-quadruplex-stabilizing molecules and CDK2, CHK1, STAT5, PI3K-δ, BRD4, BRPF1 and FLT3 inhibitors. Different strategies led to the development or discovery of these compounds: some of them were synthesized with the aim of targeting specific molecules, as for many FLT3 inhibitors, and their structure and moieties have been progressively replaced in order to improve their efficacy and selectivity. Conversely, other agents are products of synthesis showing an antitumor activity in general and/or in leukemia in particular, that have been tested against potential targets as a second step. For a minority of them, SAR and/or docking studies have not been conducted, and the cellular target still needs to be identified. This step is crucial for the definition of the molecule’s specificity and selectivity so that it can be adjusted through the synthesis of derivatives and novel or improved therapeutic combinations against acute leukemia cells can be suggested. The mechanisms of action, including the downstream intracellular changes induced by in vitro treatment, have been investigated for a number of molecules, though with different depth levels (from selective cell phenotype analysis to signaling activation or transcriptome analysis, allowing a broader view of their effects). Some of them showed promising results, with active concentrations in the nanomolar range, while others were effective at micromolar concentrations. Few compounds have also been tested on primary leukemia samples. The compounds showing a preclinical activity at lower doses, along with elevated bio-availability and lower toxicity in vivo, represent good candidates for future clinical investigation. Moreover, a deeper investigation of the transcriptional changes induced by the newly synthesized molecules is recommended, since it can uncover the spectrum of biological effects induced at the intracellular level. This knowledge is crucial to design novel effective therapeutic strategies, aiming to target multiple oncogenic mechanisms while reducing the drug doses [132] through synergic combinations.

## Figures and Tables

**Table 1:** New molecules targeting FLT3 with no available evidence on cell cycle effects.

Molecule.	Target(s)	Disease(s)	IC50 /GI50	Reference	SAR/Docking Studies
5-(4-fluorophenyl)-N-phenyloxazol-2-amine (compound **7c**)	FLT3, FLT3-ITD	AML	MV4-11: 95.51 ± 1.16 nM MOLM-13: 61.9 ± 2.45 nM	[54]	Yes
4-((6,7-dimethoxyquinoline- 4-yl)oxy)aniline derivatives (compound **12c**)	FLT3	AML	MV4-11: 8.29 ± 0.24 µM HL-60: 14.16 ± 0.22 µM	[55]	Yes
4-((6,7-dimethoxyquinoline- 4-yl)oxy)aniline derivatives (compound **12g**)	FLT3	AML	MV4-11: 5.80 ± 0.42 µM HL-60: 8.91 ± 0.66 µM	[55]	Yes
4-azaaryl-N-phenylpyrimidin-2-amine derivative (compound **12b**)	FLT3, FLT3-ITD and their mutants	AML	MV4-11: 0.074 ± 0.010 µM MOLM-13 0.023 ± 0.001 µM	[56]	Yes
4-azaaryl-N-phenylpyrimidin-2-amine derivative (compound **12r**)	FLT3, FLT3-ITD and their mutants	AML	MV4-11: 0.017 ± 0.012 µM MOLM-13: 0.0004 ± 0.0002 µM	[56]	Yes
3-aminoisoquinoline analogs (HSW630-1)	FLT3	AML	MV4-11: 0.15 µM MOLM-14: 0.15 µM	[57]	Yes
3-amino and 1-aminoisoquinoline benzamides (HSN286)	FLT3, SRC kinases	AML	MV4-11: 0.492 nM MOLM-14: 0.721 nM	[58]	Yes
N-(3,4-dimethoxybenzyl)-1-phenyl-1H-benzimidazol-5-amine derivative (compound HP1328)	FLT3, FLT3-ITD, c-KIT	AML	Ba/F3 *FLT3*-ITD: 75.4 ± 3.2 nM MV4-11: 165.0 ± 17.5 nM MOLM-13: 66.8 ± 4.8 nM	[59]	Yes
pyrimidine-4,6-diamine derivative (compound **13a**)	FLT3	AML	Ba/F3 *FLT3*-ITD: 131.2 nM MV4-11: 9.9 nM MOLM-14: 24.4 nM MOLM-14^D835Y^:1842 nM MOLM-14^F691L^: 1345 nM	[60]	Yes
amino acid-substituted sunitinib analogue released active compound candidates (**20a**)	FLT3-ITD	AML	MV4-11: 2.2 ± 0.3 µM	[61]	Yes
phenylethenylquinazoline derivatives (compound **III**)	FLT3-ITD, FLT3^D835Y^, FLT3-ITD^D835Y^	AML	MV4-11: 0.03 ± 0.00 µM	[62]	Yes
bis(1H-indol-2-yl)methanones, detivatives (compound **16**)	FLT3, FLT3-ITD, FLT3^D835Y^, RET, ZAK	AML	MV4-11: <0.001 µM MOLM-13: <0.001 µM EOL-1: <0.001 µM	[63]	Yes
(Z)-N-(5-((5-Fluoro-2-oxoindolin-3-ylidene)methyl)-4-methyl-1H-pyrrol-3-yl)-3-(pyrrolidin-1-yl)propanamide (compound **17**)	FLT3-ITD and its mutants	AML	MV4-11: 23.5 ± 1.2 nM MOLM-13: 35.5 ± 2.1 nM Ba/F3 *FLT3*-ITD: 12.7 ± 0.1 nM Ba/F3 *FLT3*-ITD^D835Y^: 36.7 ± 0.7 nM Ba/F3 *FLT3*-ITD^D835V^: 26.8 ± 1.5 nM Ba/F3 *FLT3*-ITD^F691L^: 43.6 ± 3.1 nM	[64]	Yes
imidazo[1,2-a]pyridine-thiophene derivative (compound **5o**)	FLT3 and its mutants	AML	MOLM-14: 0.52 ± 0.062 µM MOLM-14 *FLT3*-ITD^D835Y^: 0.53 ± 0.022 µM MOLM-14 *FLT3*-ITD^F691L^: 0.57 ± 0.058 µM	[65]	Yes
2-Aminopyrimidine derivative (compound **30**)	FLT3, TRKA, Aurora A	AML	MV4-11: 3.20 ± 0.77 nM Ba/F3 *FLT3*-ITD: 23.32 ± 8.27 nM Ba/F3 FLT3^D835V^: 1.41 ± 0.11 nM Ba/F3 *FLT3*^D835F^: 5.02 ± 0.87 nM Ba/F3 *FLT3*^F691L^: 28.84 ± 5.01 nM Ba/F3 *FLT3*-ITD^D835Y^: 19.23 ± 10.46 nM Ba/F3 *FLT3*-ITD^F691L^: 99.62 ± 3.22 nM	[66]	Yes
2-Aminopyrimidine derivative (compound **36**)	FLT3, TRKA, Aurora A	AML	MV4-11: 0.75 ± 0.11 nM Ba/F3 *FLT3*-ITD: 0.84 ± 0.33 nM Ba/F3 *FLT3*^D835V^: 1.29 ± 0.10 nM Ba/F3 *FLT3*^D835F^: 0.16 ± 0.03 nM Ba/F3 *FLT3*^F691L^: 3.56 ± 0.48 nM Ba/F3 *FLT3*-ITD^D835Y^: 1.71 ± 0.54 nM Ba/F3 *FLT3*-ITD^F691L^: 14.50 ± 1.02 nM	[66]	Yes
1H-pyrazole-3-carboxamide derivatives (compound **8t**)	FLT3, CDKs, KDR/VEGFR2, ERK7, FLT1, FLT4, GSK3	AML, T-ALL	MV4-11: 1.22 nM HL-60 1.15 µM CCRF-CEM: 0.22 µM MOLT-4 0.08 µM	[67]	Yes

**Table 2:** New molecules targeting FLT3 that alter cell cycle progression.

Molecule	Target(s)	Disease(s)	IC50 /GI50	Cell Cycle Arrest (Phase, Model, Dose, Time Point)	Reference(s)	SAR/Docking Studies
N-(4-(6-Acetamidopyrimidin-4-yloxy)phenyl)-2-(2-(trifluoromethyl)phenyl)acetamide (CHMFL-FLT3-335, compound **27**)	FLT3-ITD, PDGFRβ, HPK1, CSF1R, PDGFRα, c-KIT	AML	MV4-11: 0.284 ± 0.018 µM MOLM-13: 0.466 ± 0.026 µM MOLM-14: 0.343 ± 0.025 µM	G0/G1, MV4-11: 0.1–3 µM, 24 h MOLM-13: 0.1–3 µM, 12 h MOLM-14: 0.3–3 µM, 24 h	[68]	Yes
pyrrolo[2,3-d]pyrimidine derivatives (compound **9u**)	FLT3-ITD, PDGFRα, ABL1 and their mutants, HCK, LCK, RET, LYN, MET, MER, TIE2	AML	MV4-11: 0.089 ± 0.001 nM MOLM-13: 0.022 ± 0.003 nM Ba/F3 *FLT3*-ITD: 0.92 ± 0.01 nM Ba/F3 *FLT3*-ITD^D835Y^: 20.71 ± 2.32 nM Ba/F3 *FLT3*-ITD^F691L^: 12.99 ± 0.87 nM	G0/G1 MV4-11: 1–30 nM, 24 h MOLM-13: 3–30 nM, 24 h	[69]	Yes
8,9,10,11-tetrahydro-3H-pyrazolo[4,3-a]phenanthridine (HSD1169, compound **10**)	FLT3, FLT3-ITD and its mutants, LRRK2, MELK, DYRK1B, CLK1, TRKC, MNK2, TOPK, ROCK2, MSK2, p70S6K, PKG1a, CDK2/Cyclin A1	AML	MV4-11: 5.4 nM MOLM-14: 4.9 nM MOLM-13 *FLT3*-ITD^D835Y^: 5.1 nM	G1, MV4-11: 62.5 nM, 48–72 h	[70,71]	Yes
8,9,10,11-tetrahydro-3H-pyrazolo[4,3-a]phenanthridine derivative (compound **49**)	FLT3, FLT3-ITD and its mutants, LRRK2, MELK, DYRK1B, CLK1, TRKC	AML	MV4-11: 0.070 µM MOLM-13: 0.068 µM MOLM-14: 0.026 µM MOLM14 *FLT3*^D835Y^: 0.036 µM MOLM14 *FLT3*^F691L^: 0.053 µM Ba/F3 *FLT3*-ITD: 0.062 µM	G1, MV4-11: 20–500 nM, 24 h	[71]	Yes
*O*-methylated flavonol (compound **11**)	FLT3, other kinases	AML, ALL	MOLM-13: 2.65 ± 0.28 μM MV4-11: 1.99 ± 0.25 μM HL-60: 12 ± 4.39 µM MOLT-4: 7.95 ± 1.9 µM	G0/G1, MOLM-13: 3 μM, 16–72 h MV4-11: 3 μM, 16–72 h	[72]	Yes
Flavonoid derivatives (compound **31**)	FLT3, FLT3^D835Y^, FLT3-ITD	AML	MOLM-13: 2.6 ± 0.20 μM MV4-11: 2.6 ± 0.46 μM	G0/G1 MOLM-13: 3 μM, 72 h MV4-11: 3–30 μM, 72 h	[73]	Yes
Flavonoid derivatives (compound **32**)	FLT3, FLT3^D835Y^, FLT3-ITD	AML	MOLM-13: 32 5.9 ± 0.58 μM MV4-11: 7.9 ± 0.20 μM	G0/G1 MV4-11: 3–10 μM, 72 h	[73]	Yes
5,7,4′-trihydroxy-6-methoxyflavone (compound **40**)	FLT3, FLT3^D835Y^, FLT3-ITD	AML	MOLM-13: 40 7.0 ± 0.71 μM MV4-11: 6.8 ± 0.66 μM	G0/G1 MOLM-13: 3 μM, 72 h MV4-11: 3–10 μM, 72 h	[73]	Yes
Fradcarbazole A derivative (compound **6**)	FLT3, c-KIT, CDK2	AML	MV4-11: 0.32 ± 0.03 µM	G0/G1, MV4-11: 0.15–0.6 μM, 24 h	[74]	No
3-amine-pyrazole-5-benzimidazole compounds (**67**)	FLT3-ITD and its mutants	AML	MOLM-13: 9.85 ± 1.03 nM MV4-11: 2.93 ± 0.31 nM Ba/F3 *FLT3*-ITD: 7.60 ± 0.58 nM Ba/F3 *FLT3*-ITD^F691L^: 8.30 ± 0.51 nM Ba/F3 *TEL*-*FLT3*^D835V^, Ba/F3 *FLT3*-ITD^Y842H^, Ba/F3 *FLT3*-ITD^D835V^: <1.50 nM	G1, MV4-11: 10–100 nM, 24 h	[75]	Yes
6-(pyrimidin-4-yl)- 1*H*-pyrazolo[4,3-*b*]pyridine derivative (compound **23k**)	FLT3, CDK4	AML	MV4-11: 70 ± 8 nM	G0/G1, MV4-11: 200 nM, 24 h	[76]	Yes
1-H-pyrazole-3-carboxamide derivatives (compound **50**)	FLT3, CDK2,4,6	AML	MV4-11: 0.008 ± 0.001 µM	G0/G1, MV4-11: 0.02–0.2 μM, 24 h	[77]	Yes
pyrido-dipyrimidines (compound **20**)	topoisomerase II, FLT3	AML	HL-60: 0.48 ± 0.08 µM	G2/M, HL-60: 2.26 μM, 24 h	[78]	Yes
N-phenyl-4-(thiazol-5-yl)pyrimidin-2-amines and 4-(indol-3-yl)-N-phenylpyrimidin-2-amines (**16a**)	FLT3, MNK2	AML	MV4-11: 0.60 ± 0.10 µM	G1, MV4-11: 4.8 μM, 48 h	[79]	Yes

**Table 3:** New molecules targeting other proliferative signals.

Molecule	Target(s)	Disease(s)	IC50 /GI50	Reference	SAR/Docking Studies
Indole scaffold derivative (FD223, compound **13**)	PI3Kδ	AML	HL-60: 2.25 μM, MOLM-16: 0.87 μM EOL-1: 2.82 μM KG-1: 5.82 μM	[92]	Yes
PPARα/γ ligand derivative (compound **17f**)	STAT5	AML	KG1a: 2.638 ± 0.51 μM MV4-11: 3.549 ± 0.47 μM	[93]	Yes
17f analogs (compounds **7a** and **7a’**)	STAT5	AML	KG-1a: **7a**, 7.8±0.9 μM **7a’**, 6.9±0.8 μM MOLM-13: **7a**, 5.4±0.5 μM **7a’**, 4.7±1.0 μM	[94]	No
2,9-bis[4-(pyridinylalkylaminomethyl)phenyl]-1,10-phenanthroline derivatives (compound **1g**–**i**)	G- quadruplexes	AML	MV4-11: 1g, 2.1 ± 0.5 µM 1h, 1.3 ± 0.3 µM 1i, 1.6 ± 0.4 µM U937: 1 g, 2.0 ± 0.8 µM 1h, 2.0 ± 0.7 µM 1i, 3.0 ± 0.9 µM HL-60: 1 g, 3.0 ± 1.0 µM 1h, 8.0 ± 0.9 µM 1i, 3.0 ± 0.8 µM	[95]	FRET melting experiments and native electrospray mass spectrometry
biimidazole derivative (BIM-2)	G- quadruplexes	AML	U937: 9.2 μM	[96]	Yes
Quinizarin quaternary ammonium salt **3**	unknown	AML, T-ALL	HL-60: 1.40 ± 0.81 μM MOLT-4: 2.61 ± 0.15 μM Jurkat: 2.80 ± 0.22 μM	[97]	Yes
3,5-dimethylisoxazole derivatives (compound **58**)	BRD4	AML	MV4-11: 0.15 ± 0.02 µM HL-60: 1.21 ± 0.02 µM	[98]	Yes
3-Hydroxyisoindolin-1-one derivate (compound **10e**)	BRD4	AML	MV4-11: 0.420 ± 0.011 µM HL-60: 0.365 ± 0.018 µM	[99]	Yes
Quinolin-2-one Hit (compound **13-d**)	BRPF1	AML	OCI-AML2: 1.3 µM NOMO-1: 4.6 µM THP-1: 5.7 µM KG-1: 7.0 µM MV4-11: 9.9 µM	[100]	Yes

**Table 4:** New molecules targeting proliferation with unknown targets.

Molecule	Target(s)	Disease	IC50 /GI50	Reference	SAR/Docking Studies
3′, 5′-diprenylated chalcone	unknown	Erythroleukemia	HEL: 2.027 ± 0.523 µmol/L	[117]	No
dithiocarbamate esters of parthenolide (compound **7l**)	unknown	AML	KG-1a: 0.7 ± 0.2 μM. HL-60: 1.7 ± 0.5 μM.	[118]	No
Neglectin derivative (compound **8**)	unknown	AML	HL-60: 7.24 ± 0.15 µM	[119]	No
2,4-dinitrobenzenesulfonamide derivative (S1)	unknown	T-ALL	Jurkat: 6.0 ± 0.4 μM	[120]	No
Octatetraenylpyrrole (compound **3s**)	unknown	T-ALL	CCRF-CEM: 0.27 μM.	[121]	No

**Table 5:** New molecules targeting the cell cycle machinery.

Molecule	Target(s)	Disease(s)	IC50 /GI50	Reference	SAR/Docking Studies
2-carbomethoxy-3-substituted indoles (compound **20**)	Tubulin (suggested by cell phenotype)	ALL, AML	CEM: 0.22 µM RS4;11: 0.30 µM	[122]	No
Z)-2-((2-((1-ethyl-5-methoxy-1H-indol-3-yl)methylene)-3-oxo-2,3-dihydrobenzofuran-6-yl)oxy)acetonitrile (**5a**)	Tubulin	T-ALL, B-ALL	CRF-CEM: 244 nM DND41: 210 nM Jurkat: 273 nM HBP-ALL: 94 nM Loucy: 334 nM MOLT-4: 241 nM MOLT-16: 234 nM RPMI8402: 301 nM NALM-16: 272 nM REH: 287 nM	[123]	Yes
CDK2-PROTAC	CDK2	AML	MV4-11: 100 nM < IC50 < 1 µM	[124]	Yes
Diaminopyrimidine derivatives (compound **13**)	CHK1	AML	MV4-11: 0.035 ± 0.007 μM	[125]	Yes
1-substituted pyrido[4,3-b]carbazole derivatives of olivacine	TP53 restoration	T-ALL	CCFR/CEM: Compound **1**: 0.442 ± 0.062 µM Compound **2**: 0.520 ± 0.185 µM Compound **3**: 0.359 ± 0.109 µM	[126]	No

## Data Availability

Not applicable.
